# Quantification of Immediate Surgical Stress After Major and Minor Thoracic Surgeries Through HRV: A Secondary Post Hoc Analysis of a Randomized Controlled Trial

**DOI:** 10.1155/pm/3752161

**Published:** 2026-09-30

**Authors:** Seyedsadra Miri, Sabina Lähteenmäki, Heidi Mahrberg, Antti Vehkaoja, Jari Laurikka, Jari Viik

**Affiliations:** ^1^ Finnish Cardiovascular Research Center–Tampere Tampere University Tampere Finland uta.fi; ^2^ Faculty of Medicine and Health Technology Tampere University Tampere Finland uta.fi; ^3^ Tampere University Hospital Heart Center Tampere Finland

**Keywords:** autonomic nervous system, electrocardiogram, heart rate variability, physiotherapy interventions, postoperative recovery, recovery assessment

## Abstract

**Purpose:**

Heart rate variability (HRV) reflects autonomic nervous system activity and is sensitive to physiological stress induced by surgery. This study is a secondary, post hoc analysis of a randomized controlled trial aimed at evaluating changes in HRV following major and minor thoracic surgery, and investigating whether postoperative respiratory physiotherapy can modulate these changes. In a prospective, randomized design, two physiotherapy interventions, including positive expiratory pressure (PEP) and inspiratory muscle training (IMT), were compared to assess their effects on HRV.

**Methods:**

Electrocardiogram recordings were analyzed from 75 patients undergoing either minor pulmonary (PM) or pulmonary resection (PR) surgery. HRV data were collected preoperatively and during the first two postoperative days, immediately following physiotherapy sessions. Patients were assigned to one of two intervention groups (PEP or IMT). Time‐, frequency‐, and nonlinear‐domain HRV parameters were analyzed longitudinally and compared between surgical and intervention groups.

**Results:**

Most HRV parameters exhibited a postoperative reduction, with more pronounced changes observed in the PR surgery group. In particular, the root mean square of successive RR interval differences (RMSSD) and high‐frequency (HF) power, which are commonly associated with parasympathetic modulation, showed marked decreases. Recovery toward baseline values varied across surgical groups, interventions, and HRV parameters; however, no clear signs of recovery were observed in the PR surgery group during the observation period. No statistically significant differences were detected between the PEP and IMT interventions (*p* ≥ 0.05), with the exception of short‐term detrended fluctuation analysis (DFA *α*_1_) in the PM surgery group (*p* = 0.04).

**Conclusion:**

HRV parameters reflected surgery‐induced autonomic alterations, with more pronounced and persistent changes observed in patients undergoing PR compared with PM surgery. No clear recovery toward baseline was detected during the 2‐day postoperative follow‐up, suggesting prolonged autonomic disturbances after thoracic surgery.

**Trial Registration:** ClinicalTrials.gov: NCT02931617

## 1. Introduction

Major lung surgery typically involves resection of pulmonary lobes and mediastinal lymph nodes, and can substantially affect the autonomic nervous system (ANS) by disrupting the integrity of vagal nerve branches. The ANS plays a critical role in maintaining physiological homeostasis, with its sympathetic (SNS) and parasympathetic (PNS) branches acting in a coordinated and often opposing manner to regulate the functions of multiple organs and glands. Alterations in internal physiological states, whether induced by environmental influences or physiological stressors, are commonly reflected in changes in ANS activity [1]. Surgical manipulation, together with the physiological and psychological stress associated with surgery, can lead to pronounced disturbances in autonomic regulation [2]. Furthermore, the effects of general anesthesia on sympathetic activity add complexity to ANS responses during and after surgical procedures [3]. Given the central role of the ANS in regulating diverse bodily functions, monitoring its activity can provide valuable insights into a patient′s physiological status throughout the perioperative and recovery periods.

Heart rate variability (HRV) is a well‐established, noninvasive tool for assessing ANS function. In clinical settings, HRV has been used, for example, to predict postoperative complications [4–6], assess arrhythmias [7], and evaluate the effects of physiotherapy interventions following surgery [8]. HRV can be measured using various noninvasive techniques, including electrocardiography (ECG), optical heart rate (OHR) monitoring, and bed‐integrated ballistocardiography (BCG) [9–11]. HRV can be quantified using a range of linear and nonlinear parameters, each providing insight into different aspects of autonomic regulation. Although the interaction between the SNS and PNS branches of the ANS is inherently nonlinear, certain HRV parameters are more closely associated with one branch or with overall autonomic modulation. For instance, the root mean square of successive RR interval differences (RMSSD) and the high‐frequency (HF) power component of the RR interval tachogram are primarily associated with parasympathetic modulation. However, as the HF band is also influenced by respiratory rate, interpretations based solely on parasympathetic function should be made with caution. In contrast, SDNN and low‐frequency (LF) power reflect broader aspects of autonomic modulation, whereas nonlinear HRV measures characterize the complexity and temporal organization of the RR interval time series [12, 13].

Previous studies have demonstrated that reduced HRV is associated with an increased risk of postoperative complications. For example, Ernst et al. [14] reported that lower preoperative HRV, reflected by reduced RMSSD and total power, predicted postoperative complications in patients with hip fractures. Similarly, patients undergoing digestive surgery who developed postoperative complications exhibited lower HRV values, supporting the role of HRV as an indicator of surgical stress and impaired autonomic regulation [15]. In addition, HRV parameters have been evaluated as preoperative risk classifiers for cardiovascular complications and autonomic dysfunction [16]. However, relatively few studies have investigated the potential of HRV as a monitoring tool in adult pulmonary surgery, where extensive pulmonary manipulation and pronounced autonomic stress may substantially influence postoperative recovery, particularly in elderly patients.

This study is aimed at addressing this gap by investigating whether ECG‐derived HRV parameters can be used to assess the severity of and quantify the surgical stress associated with pulmonary surgeries of different extents, including major procedures involving lobar or larger resections, and minor procedures such as lung biopsies, where tissue loss is limited. In addition, two physiotherapy interventions including positive expiratory pressure (PEP) and inspiratory muscle training (IMT) were implemented in a randomized manner across both surgical groups (pulmonary resection [PR] and minor pulmonary [PM] surgery) to promote postoperative recovery. We further aim to evaluate the short‐term effectiveness of these interventions through HRV‐derived changes. PEP has been shown to enhance lung volumes, improve airway clearance, and reduce hyperinflation [17], whereas IMT improves inspiratory muscle strength and endurance [18, 19]. In addition, a recent study reported that IMT improved expiratory function in patients undergoing thoracotomy [20]. By assessing ANS responses and recovery trajectories over a 2‐day period following surgery, this study seeks to determine whether HRV can serve as an effective monitoring tool for postoperative recovery in pulmonary surgery patients. We hypothesized that HRV parameters will reflect short‐term differences in recovery between surgical extents and between the two physiotherapy interventions.

## 2. Materials and Methods

### 2.1. Study Participants

This study is a secondary, post hoc analysis of data obtained from a randomized controlled trial (RCT) conducted at Tampere University Hospital between 2013 and 2016, including patients undergoing cardiac surgery, minor thoracic surgery, and major thoracic surgery. The present analysis focuses on HRV responses in relation to surgical extent and intervention groups among thoracic surgery patients.

A total of 84 patients undergoing either PM (*n* = 44) or PR surgery (*n* = 40) were included in this secondary analysis. PR surgery included major anatomical lung resections such as lobectomy, bilobectomy, and pneumonectomy. PM procedures included less extensive interventions with limited impact on lung volume, such as wedge resections, lung biopsies, and other minor pleuropulmonary procedures (e.g., excisions or enucleations). Classification was based on the extent of lung tissue removal and expected physiological impact. A detailed list of surgical procedures is provided in the Table S1. Patients were randomized into two respiratory physiotherapy intervention groups: PEP and IMT, which were applied postoperatively to promote recovery. In the PM group, 25 patients received PEP and 19 received IMT, whereas in the PR group, 19 patients received PEP and 21 received IMT.

Ten‐min resting ECG recordings were obtained preoperatively (PREOP) and on the first two postoperative days (POD1 and POD2), immediately following the standardized physiotherapy sessions. Only participants with complete and analyzable HRV data across all study time points were included in the final analysis (PM–PEP, *n* = 19; PM–IMT, *n* = 18; PR–PEP, *n* = 18; PR–IMT, *n* = 20). All participants provided written informed consent, and the study protocol was approved by the Regional Ethics Committee of Tampere University Hospital (R13037). All procedures were conducted in accordance with the principles of the Declaration of Helsinki.

Study variables are presented in Tables 1 and 2. Baseline characteristics included age, sex, BMI, weight, and comorbidities such as chronic obstructive pulmonary disease (COPD), coronary artery disease (CAD), hypertension (HTA), type 2 diabetes (DM2), asthma, and asbestosis. Table 1 presents the randomized comparison between PEP and IMT intervention groups, stratified by surgical subgroup (PM and PR), whereas Table 2 shows the observational comparison between the PM and PR surgical groups, independent of intervention assignment. Continuous variables are presented as mean ± standard deviation and were compared using the independent samples *t*‐test. Categorical variables are presented as counts and were compared using Fisher′s exact test. A *p* value < 0.05 was considered statistically significant.

**Table 1:** Baseline characteristics of the study population, presented as a randomized comparison between PEP and IMT intervention groups, stratified by surgical subgroup (PM and PR).

Variables	PM (minor) surgery group			PR (major) surgery group		
	PEP (*n* = 19)	IMT (*n* = 18)	*p*	PEP (*n* = 18)	IMT (*n* = 20)	*p*
Age (year)	56.67 ± 15.71	57.68 ± 11.19	0.821	68.67 ± 8.81	66.32 ± 5.76	0.340
Sex (male/female)	9/10	9/9	1.000	13/5	11/9	0.495
BMI (kg m^−2^)	27.88 ± 5.96	29.23 ± 6.70	0.559	27.61 ± 3.84	27.11 ± 5.56	0.750
Weight (kg)	81.27 ± 19.48	70.86 ± 27.60	0.196	81.83 ± 14.57	77.87 ± 16.08	0.438
COPD (*n*/total)	2/19	1/18	0.603	4/18	6/20	0.714
CAD (*n*/total)	1/19	0/18	0.486	2/18	2/20	1.000
HTA (*n*/total)	6/19	8/18	0.737	10/18	7/20	0.330
DM2 (*n*/total)	2/19	1/18	0.603	6/18	1/20	**0.042**
Asthma (*n*/total)	0/19	2/18	0.486	3/18	3/20	1.000
Asbestosis (*n*/total)	1/19	1/18	1.000	2/18	2/20	1.000

*Note:* Continuous variables are reported as mean ± standard deviation (SD), and categorical variables as counts. Statistically significant values are highlighted in bold (*p* < 0.05).

**Table 2:** Baseline characteristics of the study population, presented as an observational comparison between the PM and PR surgical groups, independent of intervention assignment.

Variables	PM (minor) surgery group (*n* = 37)	PR (major) surgery group (*n* = 38)	*p*
Age (year)	57.19 ± 13.39	67.46 ± 7.40	**< 0.001**
Sex (male/female)	18/19	24/14	0.240
BMI (kg m^−2^)	28.45 ± 6.21	27.35 ± 4.74	0.411
Weight (kg)	75.92 ± 24.25	79.80 ± 15.28	0.413
COPD (*n*/total)	3/37	10/38	0.640
CAD (*n*/total)	1/37	4/38	0.357
HTA (*n*/total)	14/37	17/38	0.637
DM2 (*n*/total)	3/37	7/38	0.308
Asthma (*n*/total)	2/37	6/38	0.261
Asbestosis (*n*/total)	2/37	4/38	0.674

*Note:* Continuous variables are reported as mean ± standard deviation (SD), and categorical variables as counts. Statistically significant differences between groups are indicated in bold (*p* < 0.05).

### 2.2. ECG Data Acquisition

ECG measurements were collected using a portable device comprising a measurement unit and a control and communication unit, with signals sampled at 256 Hz [21]. The measurement unit is designed for continuous ambulatory recording of physiological signals and integrates dedicated modules for ECG alongside other biosignals. Disposable ECG electrodes were positioned on both sides, along the midaxillary line, near the armpits, and 10‐min recordings were taken during noncontrolled tidal breathing [22]. To ensure data quality and consistency, participants who did not have complete recordings for the required days (PREOP, POD1, or POD2) or whose ECG files contained excessive artifacts, making accurate R peak detection impossible, were excluded from the analysis (*n* = 9). Patients were receiving their normal necessary medications for pain and for other indications during the study.

### 2.3. ECG Data Preprocessing

The ECG recordings were initially filtered to remove baseline wander and HF noise caused by power‐line interference. Filtering was performed using a median filter with a 100‐ms window and a low‐pass filter with a 40‐Hz cutoff frequency. R‐peaks were then detected using a differential threshold‐ and amplitude‐based method as proposed by Zhengzhong et al. [23]. R‐peak locations were identified at the original ECG sampling resolution (256 Hz), and RR intervals were computed directly from the detected R‐peak indices without temporal interpolation. After construction of the RR tachogram, ectopic beats were removed using the method proposed by Lipponen and Tarvainen [24], which applies adaptive thresholds based on the time‐varying distribution of RR intervals to identify ectopic and misdetected beats while preserving normal sinus rhythm. All analyses were conducted using MATLAB R2019b. Table 3 summarizes the nine commonly used linear and nonlinear HRV parameters evaluated in this study.

**Table 3:** Description of the evaluated HRV parameters.

Parameters [unit]	
Time domain parameters	
SDNN [ms]	Standard deviation of NN intervals
RMSSD [ms]	Root mean square of successive differences
Mean HR [bpm]	Mean of heart rates
Frequency domain parameters	
VLF abs [ms^2^]	Very low‐frequency absolute power (0.0033 < VLF < 0.04 Hz)
LF abs [ms^2^]	Low‐frequency absolute power (0.04 < LF < 0.15 Hz)
HF abs [ms^2^]	High‐frequency absolute power (0.15 < HF < 0.4 Hz)
Nonlinear parameters	
DFA *α*_1_ [‐]	Short‐term fluctuation slope
ApEn [‐]	Approximate entropy
SD_2_/SD_1_ [‐]	Poincaré plot long‐term variability/Poincaré plot short‐term variability

### 2.4. HRV Analysis

Several linear and nonlinear HRV parameters were selected to provide a comprehensive understanding of different aspects of ANS activity. Time‐domain (e.g., SDNN and RMSSD), frequency‐domain (e.g., LF, HF, and VLF), and nonlinear HRV measures each describe different aspects of HR dynamics and collectively provide complementary information on autonomic regulation. Poincaré plot analysis was included because it provides quantitative descriptors of beat‐to‐beat variability, with SD_1_ reflecting short‐term variability primarily related to rapid beat‐to‐beat fluctuations, and SD_2_ reflecting longer term variability in the Poincaré plot. Approximate entropy (ApEn) quantifies the regularity and complexity of the RR interval time series, whereas short‐term detrended fluctuation analysis (DFA *α*_1_) characterizes short‐term fractal correlation properties of HR dynamics. Together, these nonlinear measures complement conventional time‐ and frequency‐domain HRV analyses by providing additional information regarding the organization and complexity of cardiac dynamics. However, the activities of the SNS and PNS cannot be clearly separated using individual HRV parameters, as autonomic regulation is complex and influenced by multiple physiological mechanisms [13]. The HRV parameters analyzed in this study are described in Table 3.

### 2.5. Statistical Analysis

The statistical significance of changes in HRV parameters across different surgical days and between interventions was evaluated. The normality of the datasets was first assessed using the Shapiro–Wilk test. For normally distributed data, paired comparisons within the same participants were assessed using the paired‐samples *t*‐test, whereas the Wilcoxon signed‐rank test was used for nonnormally distributed paired data. For comparisons between independent groups, the independent‐samples *t*‐test was used for normally distributed data, whereas the Wilcoxon rank‐sum test was used for nonnormally distributed data. To account for potential confounding due to age differences between the PM and PR groups, an analysis of covariance (ANCOVA) was performed, with age included as a covariate in the linear model. In this study, statistical significance was considered at three levels: *p* ≤ 0.05 (∗), *p* ≤ 0.01 (∗∗), and *p* ≤ 0.005 (∗∗∗). All analyses were performed using MATLAB R2019b.

To further complement the evaluation of surgical effects and the comparison between PEP and IMT interventions, the postoperative values of each participant were normalized with respect to their own preoperative values. This approach enabled a more consistent comparison between interventions by accounting for interparticipant variability in individual HRV measures. The normalized values were calculated using Equation (1).

(1) HRVn normalized=PODxn−PreopnPreopn×100,

where *n* denotes the participant, and *x* corresponds to the postoperative day (1 or 2).

In the trend analysis of HRV parameters across different measurement days (PREOP, POD1, and POD2), the standard deviation is displayed alongside the mean values.

## 3. Results

### 3.1. Baseline Characteristics

A total of 84 participants were initially recruited. Of these, nine participants were excluded due to incomplete recordings or excessive artifacts following randomization. Ultimately, 75 participants were included in the analysis, comprising 37 in the PM surgery group and 38 in the PR surgery group. The participant flow and exclusion criteria are illustrated in Figure 1.

**Figure 1:**
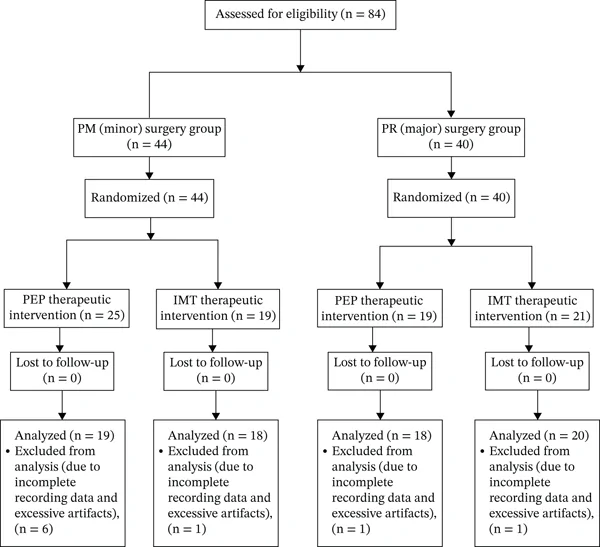
Flowchart of randomized participants. Participants were excluded due to reasons such as incomplete recording data and excessive artifacts.

The follow‐up time for each participant in the study was 3 days: PREOP, POD1 and POD2. The data collection was consistent across all participants, and no additional follow‐up was conducted beyond these time points.

### 3.2. Randomized Comparison of PEP and IMT Interventions

The per‐participant mean ± standard deviation of HRV parameters for the PM and PR surgery groups, stratified by PEP and IMT interventions, are presented in Tables 4 and 5. No statistically significant differences were observed between the PEP and IMT interventions, except for DFA *α*_1_ in the PM subgroup (*p* = 0.04).

**Table 4:** Per‐participant mean ± standard deviation of the investigated HRV parameters in the PM subgroup.

Parameter [unit]	Intervention	PREOP	POD1	POD2	IMT versus PEP *p*
Time domain parameters					
SDNN [ms]	PEP	36.84 ± 12.27	33.74 ± 10.78	33.68 ± 22.27	0.86
	IMT	41.15 ± 21.21	32.67 ± 8.91	32.35 ± 14.89	
RMSSD [ms]	PEP	18.08 ± 6.86	20.07 ± 8.62	**16.03 ± 10.88** ∗	0.43
	IMT	21.07 ± 9.64	19.75 ± 8.40	**16.38 ± 7.34** ∗	
Mean HR [bpm]	PEP	73.07 ± 11.46	71.09 ± 12.75	75.62 ± 12.35	0.83
	IMT	68.77 ± 13.44	67.20 ± 7.28	70.69 ± 8.13	
Frequency domain parameters					
VLF [ms^2^]	PEP	108.62 ± 114.18	111.19 ± 97.55	66.92 ± 52.21	0.81
	IMT	127.90 ± 98.27	80.24 ± 58.63	90.02 ± 68.73	
LF [ms^2^]	PEP	294.78 ± 155.11	**219.92 ± 131.64** ∗∗	**165.53 ± 99.90** ∗∗∗	0.31
	IMT	306.72 ± 206.87	244.50 ± 154.00	237.04 ± 203.30	
HF [ms^2^]	PEP	107.04 ± 110.70	141.74 ± 123.00	98.81 ± 125.58	0.14
	IMT	131.48 ± 113.40	130.16 ± 128.74	75.73 ± 68.17	
Nonlinear parameters					
DFA *α*_1_ [‐]	PEP	1.39 ± 0.18	**1.24 ± 0.20** ∗∗∗	1.35 ± 0.25	**0.04** ∗
	IMT	1.30 ± 0.19	1.22 ± 0.17	1.35 ± 0.20	
ApEn [‐]	PEP	1.28 ± 0.22	**1.37 ± 0.19** ∗	1.24 ± 0.33	0.98
	IMT	1.33 ± 0.22	1.43 ± 0.17	1.32 ± 0.23	
SD_2_/SD_1_ [‐]	PEP	4.29 ± 1.60	**3.55 ± 1.27** ∗	4.75 ± 3.22	0.79
	IMT	3.90 ± 1.42	3.40 ± 0.94	4.03 ± 1.48	

*Note:* Within‐participant comparisons were performed between PREOP and postoperative days (POD1 and POD2). The randomized comparison between PEP and IMT interventions was based on PREOP‐to‐POD2 changes. Statistical significance is indicated by asterisks (∗*p* < 0.05, ∗∗*p* < 0.01, and ∗∗∗*p* < 0.005). Bold values indicate statistically significant differences (*p* < 0.05) in the corresponding comparison.

**Table 5:** Per‐participant mean ± standard deviation of the investigated HRV parameters in the PR subgroup.

Parameter [unit]	Intervention	PREOP	POD1	POD2	IMT versus PEP *p*
Time domain parameters					
SDNN [ms]	PEP	32.58 ± 13.71	**24.15 ± 11.38** ∗	**22.82 ± 10.34** ∗∗∗	0.23
	IMT	38.81 ± 12.90	**29.20 ± 16.33** ∗	**23.89 ± 11.36** ∗∗∗	
RMSSD [ms]	PEP	17.97 ± 9.68	15.04 ± 9.45	14.54 ± 9.88	0.27
	IMT	18.25 ± 6.80	**13.54 ± 6.48** ∗	**11.37 ± 6.16** ∗∗∗	
Mean HR [bpm]	PEP	70.26 ± 12.45	71.53 ± 12.65	74.40 ± 15.06	0.82
	IMT	68.79 ± 10.12	69.90 ± 10.38	72.63 ± 9.33	
Frequency domain parameters					
VLF [ms^2^]	PEP	103.10 ± 96.75	**37.24 ± 24.85** ∗∗∗	**37.39 ± 32.38** ∗∗∗	0.70
	IMT	120.77 ± 95.41	89.31 ± 107.68	**63.29 ± 102.66** ∗∗	
LF [ms^2^]	PEP	206.56 ± 146.26	**102.72 ± 79.77** ∗∗	**105.11 ± 128.21** ∗	0.08
	IMT	341.60 ± 210.08	**186.30 ± 244.54** ∗∗	**146.28 ± 218.68** ∗∗∗	
HF [ms^2^]	PEP	86.28 ± 75.45	59.57 ± 81.26	**71.03 ± 140.56** ∗	0.40
	IMT	92.27 ± 61.23	**46.48 ± 50.47** ∗	**35.42 ± 50.49** ∗∗∗	
Nonlinear parameters					
DFA *α*_1_ [‐]	PEP	1.31 ± 0.20	**1.17 ± 0.30** ∗	**1.18 ± 0.27** ∗	0.22
	IMT	1.38 ± 0.14	1.30 ± 0.25	1.34 ± 0.24	
ApEn [‐]	PEP	1.33 ± 0.19	1.35 ± 0.25	1.28 ± 0.29	0.91
	IMT	1.29 ± 0.19	1.22 ± 0.25	1.24 ± 0.29	
SD_2_/SD_1_ [‐]	PEP	3.80 ± 1.18	3.48 ± 1.65	3.45 ± 1.64	0.37
	IMT	4.26 ± 1.27	4.31 ± 1.76	4.30 ± 1.71	

*Note:* Within‐participant comparisons were performed between PREOP and postoperative days (POD1 and POD2). The randomized comparison between PEP and IMT interventions was based on PREOP‐to‐POD2 changes. Statistical significance is indicated by asterisks (∗*p* < 0.05, ∗∗*p* < 0.01, and ∗∗∗*p* < 0.005). Bold values indicate statistically significant differences (*p* < 0.05) in the corresponding comparison.

Figures 2 and 3 illustrate the trends in HRV parameters across the study period, including the preoperative and postoperative days. For each day, individual HRV values were averaged, and the standard deviation is represented by the error bars. The size of the error bars reflects the degree of interparticipant variability, with larger error bars indicating greater variability in the observed HRV values. In most cases, the trend lines for the PEP and IMT interventions run in parallel, suggesting broadly similar responses to postoperative stress and recovery. However, some deviations from this overall pattern were observed. Specifically, VLF in the minor surgery group and ApEn and HF in the major surgery group exhibited less parallel trends between the PEP and IMT interventions.

**Figure 2:**
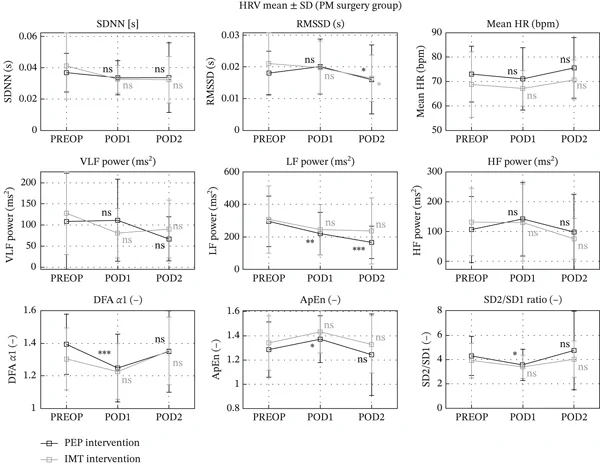
Mean ± standard deviation of HRV parameters across surgical days in the PM subgroup, stratified by intervention (PEP and IMT). Significant within‐group differences between the preoperative day (PREOP) and postoperative days (POD1 and POD2) are indicated with asterisks: *p* < 0.05 (∗), *p* < 0.01 (∗∗), and *p* < 0.005 (∗∗∗). “ns” denotes no significant difference.

**Figure 3:**
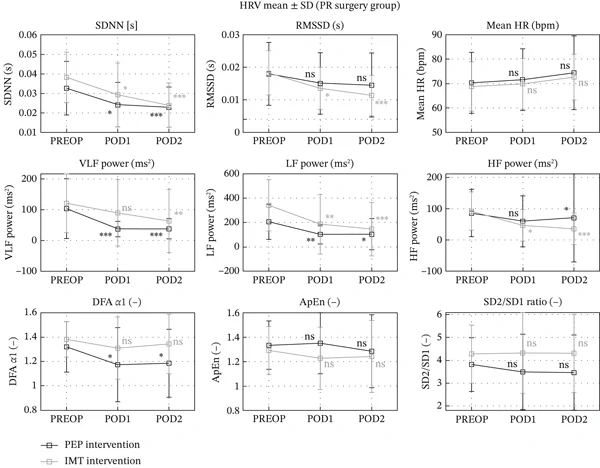
Mean ± standard deviation of HRV parameters across surgical days in the PR subgroup, stratified by intervention (PEP and IMT). Significant within‐group differences between the preoperative day (PREOP) and postoperative days (POD1 and POD2) are indicated with asterisks: *p* < 0.05 (∗), *p* < 0.01 (∗∗), and *p* < 0.005 (∗∗∗). “ns” denotes no significant difference.

Figures 4 and 5 present boxplots of POD1 and POD2 values normalized to each participant′s PREOP values for the PM and PR surgery groups, respectively. The horizontal line within each box represents the median, whereas the lower and upper edges indicate the 25th and 75th percentiles of the data distribution. Outliers are shown as individual dots. These normalized values enable side‐by‐side comparison of the physiotherapy interventions (PEP: dark gray; IMT: light gray) while accounting for interparticipant variability and differences in baseline HRV values. This presentation provides insight into surgery‐related autonomic stress, early postoperative recovery patterns, and potential differences between interventions. Statistical comparisons between PEP and IMT interventions on POD2 did not show significant differences for most parameters, with the exception of DFA *α*_1_ in the PM surgery group (*p* = 0.037).

**Figure 4:**
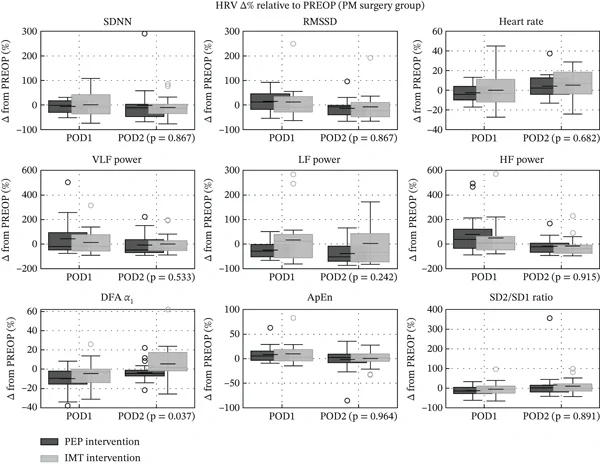
Boxplots of POD1 and POD2 values normalized to PREOP in the PM subgroup, comparing the two intervention groups (PEP, dark gray; IMT, light gray). The horizontal lines within each box represent the median, whereas the lower and upper edges correspond to the 25th and 75th percentiles, respectively.

**Figure 5:**
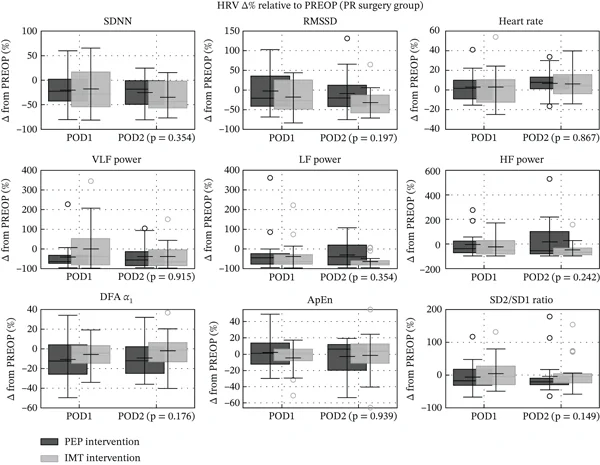
Boxplots of POD1 and POD2 values normalized to PREOP in the PR subgroup, comparing the two intervention groups (PEP, dark gray; IMT, light gray). The horizontal lines within each box represent the median, whereas the lower and upper edges correspond to the 25th and 75th percentiles, respectively.

### 3.3. Observational Comparison Between PM and PR Surgery Groups

Table 6 presents the mean ± standard deviation of the differences between PREOP and POD1 values for all included participants, together with the results of the unadjusted and age‐adjusted statistical analyses. The aim was to examine whether HRV changes differed between the PM and PR surgery groups postoperatively, reflecting the varying extent of surgery. Significant differences were observed for RMSSD and HF power in the unadjusted analysis. After adjustment for age, additional significant between‐group differences were observed for ApEn and the SD_2_/SD_1_ ratio.

**Table 6:** Mean ± standard deviation of differences of PREOP versus POD1 between the PM and PR surgery groups.

Parameter [unit]	PR (*N* = 38)	PM (*N* = 37)	*p*	Age‐adjusted *p*
SDNN [ms]	−8.70 ± 15.74	−5.71 ± 18.41	0.15	0.62
RMSSD [ms]	−3.87 ± 9.60	0.37 ± 8.41	**0.04**	**0.01**
Mean HR [bpm]	1.18 ± 10.78	−1.78 ± 9.93	0.21	0.10
VLF [ms^2^]	−47.75 ± 122.22	−21.86 ± 129.51	0.13	0.78
LF [ms^2^]	−130.92 ± 202.61	−68.69 ± 145.46	0.05	0.07
HF [ms^2^]	−36.75 ± 87.34	17.17 ± 112.25	**0.02**	**0.01**
DFA *α*_1_ [‐]	−0.10 ± 0.22	−0.11 ± 0.19	0.91	0.07
ApEn [‐]	−0.02 ± 0.24	0.08 ± 0.19	0.06	**0.001**
SD_2_/SD_1_ [‐]	−0.12 ± 1.48	−0.62 ± 1.37	0.13	**0.002**

*Note:* Statistically significant values are highlighted in bold (*p* < 0.05).

Consistent with the stratified results presented in Tables 4 and 5, postoperative differences between surgical groups were evident in HRV responses. When comparing the PM and PR groups independently of intervention assignment, the PR group showed statistically significant changes in the majority of HRV parameters from PREOP to POD1. In contrast, the PM group exhibited significant changes in fewer parameters and generally to a lesser extent, including DFA *α*_1_ and LF power. Furthermore, in the PR group, the reductions observed on POD1 did not return toward baseline levels by POD2, with HRV parameters either remaining altered or showing further deviation from PREOP values. In the PM group, some parameters (e.g., ApEn and DFA *α*_1_) that were significantly altered on POD1 were no longer significantly different on POD2, whereas others (e.g., RMSSD and LF power) showed variable recovery patterns. These group‐level patterns are also reflected in the normalized boxplots, where median HRV values tended to be lower on POD1 in the PR group compared with the PM group.

## 4. Discussion

### 4.1. Overall Postoperative Autonomic Response to Pulmonary Surgery

Pulmonary surgeries, even when limited in extent, impose substantial physiological stress on the cardiovascular and respiratory systems. Larger lung resections, such as lobectomies, may impose greater physiological stress and potentially affect autonomic regulation. Based on this physiological rationale, we hypothesized that HRV would be influenced by the extent of surgery (PR vs. PM) and/or by the type of postoperative respiratory physiotherapy intervention.

Overall, several of the investigated HRV parameters exhibited significant postoperative alterations, likely reflecting the combined effects of surgical stress and prolonged anesthesia [25]. As recovery of autonomic function has been reported to occur gradually over a period of 6–12 weeks, potentially leading to partial restoration of HRV measures [26], the postoperative changes observed in the present study should not automatically be interpreted as indicators of cardiovascular complications or increased mortality risk, but rather as potentially reflecting expected physiological responses to surgery. In addition, the observed postoperative changes in HRV may be partially attributable to transient changes in pulmonary mechanics associated with cessation of mechanical ventilation. Mechanical ventilation and its discontinuation alter intrathoracic pressures, tidal volumes, and cardiovascular–respiratory interactions, which may in turn influence autonomic regulation and HRV measures [27].

### 4.2. Comparison With Previous Studies and Clinical Relevance

HRV has been increasingly used in clinical studies as a noninvasive marker for perioperative physiological stress and recovery assessment [28]. In the context of thoracic surgery, previous studies have also reported significant alterations in HRV following surgical intervention. For example, Budgell and Polus [29] demonstrated that thoracic manipulation can lead to significant changes in frequency‐domain HRV measures, including absolute and normalized LF components as well as the LF/HF ratio. Similarly, He et al. [30] reported significant postoperative changes in both time‐ and frequency‐domain HRV parameters following pulmonary lobectomy, indicating altered autonomic regulation during the early recovery period. These findings are consistent with the present study, which also demonstrates postoperative changes in HRV‐related measures following pulmonary surgery.

### 4.3. Effect of Surgical Extent on HRV Recovery

In the PM surgery group, where only a small portion of lung tissue was removed, the mean HR decreased on POD1 and then increased on POD2, returning toward baseline levels. In contrast, in the PR surgery group, which underwent more extensive lung resection, mean HR increased postoperatively and continued to rise on POD2. This pattern suggests that the extent of surgery may have influenced HR responses differently between the two groups. The continuous HR increase in the PR surgery group may be related to more severe surgical manipulation associated with lung resection. Conversely, the transient decrease in HR in the PM group may reflect an earlier stabilization of cardiac autonomic regulation following less invasive surgery. However, factors that may influence HR, such as blood hemoglobin concentration (B‐Hb) or partial oxygen pressure (pO_2_), were not controlled for or recorded in the present study. A similar pattern was observed in the SDNN trends, which remained lower but relatively stable in the PM surgery group (Figures 2 and 3). In contrast, the PR surgery group showed a significant deviation from baseline levels during POD1 and POD2, consistent with the findings of Frandsen et al. [31], who reported that SDNN remained significantly reduced for up to 8 days following enhanced recovery after surgery (ERAS) video‐assisted thoracic surgery (VATS) lobectomy.

The results in Table 6, comparing the PM and PR surgery groups, indicate that the extent of surgery was associated with significant between‐group differences in postoperative changes in RMSSD and HF power. Both parameters are traditionally considered to reflect PNS activity [14]; however, they are also influenced by respiratory rate because respiration affects the same frequency range used to derive these measures. One possible explanation for the observed differences is that more extensive procedures, such as lobectomies, may impose greater physiological stress and may also affect autonomic pathways, including vagal nerve branches involved in cardiopulmonary regulation. Surgical dissection and lymphadenectomy performed during major resections have been associated with postoperative alterations in autonomic modulation, potentially reflected in HRV changes [32, 33]. In contrast, such extensive effects on autonomic regulation may be less likely following PM procedures, which may partly explain the observed between‐group differences. Because the PR surgery group was older than the PM surgery group, additional analyses were performed with age included as a covariate. The differences in RMSSD and HF remained statistically significant after age adjustment, suggesting that these findings cannot be explained solely by age differences between the groups. In addition, age adjustment resulted in significant between‐group differences for ApEn and the SD_2_/SD_1_ ratio, suggesting that age was associated with variation in these nonlinear HRV measures and that accounting for age revealed group differences that were not apparent in the unadjusted analysis.

In both the PM and PR surgery groups, several HRV parameters showed statistically significant differences on POD1 compared with baseline values; however, these changes were more pronounced in the PR surgery group. Among the parameters exhibiting significant POD1 alterations (e.g., SDNN, VLF, LF, and HF power), none demonstrated clear recovery toward baseline by POD2. In the PM surgery group, similar postoperative trends were observed for some parameters (e.g., LF and RMSSD); however, partial normalization by POD2 was evident for certain nonlinear indices, such as ApEn and the SD_2_/SD_1_ ratio. These findings suggest that nonlinear HRV measures may provide complementary information regarding early changes in cardiac autonomic regulation that are not fully captured by conventional linear indices [34]. Nevertheless, given the short postoperative observation period and the heterogeneity of recovery patterns across parameters, these changes should not be interpreted as definitive evidence of postoperative recovery. The more pronounced and persistent alterations in HRV observed in the PR surgery group are consistent with previous studies indicating that the magnitude of autonomic disturbance may increase with the extent of surgical trauma and pulmonary tissue resection [35].

### 4.4. Effect of Respiratory Physiotherapy Interventions

In the PM surgery group, the nonlinear parameter DFA *α*_1_ differed significantly between the PEP and IMT interventions, as indicated by the statistical analysis presented in Table 4. The normalized boxplots show that, in the PM surgery group, DFA *α*_1_ values differed between the PEP and IMT interventions on POD2, with the PEP group exhibiting a slightly more gradual return toward baseline. Although this finding suggests an intervention‐specific difference in the short‐term response of DFA *α*_1_, it does not indicate a clear superiority of either physiological intervention. Notably, this was the only HRV parameter demonstrating a statistically significant difference between PEP and IMT in the PM surgery group, and therefore the finding should be interpreted with caution. Overall, the normalized boxplots (Figures 4 and 5) demonstrate larger relative postoperative deviations in several HRV parameters in the PR surgery group compared with the PM surgery group, complementing the longitudinal trend analyses (Figures 2 and 3). This pattern suggests a more pronounced postoperative disturbance in cardiac autonomic regulation following PR. The greater magnitude of this response may be related to the extent of surgical trauma and the associated physiological demands of major PR. Consequently, the greater postoperative disturbance observed in the PR surgery group may have limited the ability of either respiratory physiotherapy intervention to produce detectable short‐term changes in HRV.

### 4.5. Interpretation of Linear and Nonlinear HRV Measures

Linear HRV measures, including SDNN, RMSSD, and frequency‐domain indices, primarily describe the magnitude and spectral distribution of HR fluctuations, whereas nonlinear measures provide complementary information regarding the complexity and temporal organization of cardiac dynamics. In the context of surgical stress, nonlinear HRV measures may provide additional insight into alterations in autonomic regulation. For example, reduced fractal‐like HR dynamics following cardiac surgery have been associated with prolonged postoperative intensive care unit (ICU) stays, suggesting that these measures may capture clinically relevant aspects of postoperative recovery beyond conventional linear HRV indices [36].

In the present study, one notable observation was made in the PM group, where the nonlinear parameters demonstrated significant postoperative changes within the PEP intervention group, whereas no significant difference from baseline was observed on POD2. This pattern may suggest a more rapid normalization of autonomic regulation following PEP intervention. Consistent with this observation, the comparison of recovery trends between interventions (Table 4) identified a significant difference in DFA *α*_1_ between the PEP and IMT groups within the PM surgery cohort. Previous studies have also suggested that modified DFA‐based approaches may improve the detection of subtle autonomic alterations in clinical populations [37–39]. Nevertheless, apart from this finding, nonlinear measures generally supported rather than substantially extended the conclusions derived from the linear HRV parameters. Furthermore, nonlinear metrics should be interpreted cautiously, as their physiological correlates remain less clearly established than those of conventional measures such as RMSSD.

### 4.6. Physiological Interpretation and Limitations of HRV Measures

Among the evaluated HRV parameters, RMSSD and HF provided the clearest evidence of postoperative autonomic alterations. The reductions observed in these parameters were more pronounced in the PR surgery group, particularly among patients receiving IMT, and remained evident on POD2, suggesting a prolonged reduction in parasympathetic modulation following more extensive surgical procedures. RMSSD is widely regarded as a robust marker of short‐term vagally mediated HR fluctuations, whereas HF power reflects respiratory sinus arrhythmia and is therefore influenced by respiratory rate and breathing pattern in addition to vagal activity [13]. Although RMSSD and HF exhibit substantial physiological overlap under resting conditions, both measures were retained in the present study because the patient population underwent pulmonary surgery, where respiratory‐related modulation of HRV may provide clinically relevant information beyond that captured by RMSSD alone. The consistent findings observed for both parameters strengthen the interpretation that PR is associated with greater postoperative autonomic disturbance than PM procedures.

Although the inclusion of multiple linear and nonlinear HRV parameters was intended to characterize different aspects of autonomic regulation, the physiological interpretation of these measures requires caution. HRV indices are indirect markers of autonomic function and should not be interpreted as direct measures of SNS and PNS activity. In particular, the physiological significance of several frequency‐domain and nonlinear parameters remains incompletely understood. LF power is influenced by multiple physiological mechanisms, including baroreflex function, parasympathetic activity, respiratory influences, and sympathetic modulation, depending on the recording conditions. Likewise, the physiological origins of VLF remain uncertain and are likely influenced by multiple regulatory processes [13]. Consequently, changes in LF or VLF should not be interpreted as direct evidence of altered sympathetic activity alone. Similarly, nonlinear indices such as DFA *α*_1_, ApEn, and the SD_2_/SD_1_ ratio provide information regarding the complexity and organization of HR dynamics; however, their physiological underpinnings remain less well established than those of conventional time‐domain measures. Therefore, although these parameters provide valuable complementary information regarding postoperative autonomic regulation, they should be regarded as supportive descriptors of HRV dynamics rather than definitive markers of specific autonomic pathways, and their physiological interpretation requires caution [40, 41].

### 4.7. Limitations

The follow‐up period in this study was relatively short, as many patients were discharged home or transferred to other departments within 3–4 days after surgery, limiting the ability to assess longer term autonomic recovery. In addition, the potential effects of perioperative medications, including postoperative analgesics and anesthetic agents, on HRV parameters could not be controlled for, as these treatments were clinically necessary and therefore represent potential source of confounding. Furthermore, the effects of the respiratory physiotherapy interventions (PEP and IMT) on HRV may have been influenced by individual patient characteristics, including baseline autonomic status and cardiovascular condition, which were not explicitly accounted for in the present analysis. Finally, a relatively large number of statistical tests were performed across multiple HRV parameters and time points, which may have increased the risk of Type I error. No formal correction for multiple comparisons was applied; therefore, the reported significant findings, particularly those with borderline significance, should be interpreted with caution.

## 5. Conclusions

HRV parameters reflected postoperative changes in cardiac autonomic regulation, with more pronounced and persistent changes observed in the PR surgery group compared with the PM surgery group, highlighting the greater physiological impact of extensive PRs. Within the short 2‐day postoperative observation period, no clear recovery toward baseline levels was detected. No statistically significant differences were observed between the PEP and IMT interventions for most HRV parameters, with the exception of DFA *α*_1_ in the PM surgery group. These findings suggest that any intervention‐specific effects on HRV may be difficult to detect during the early postoperative period, particularly following more extensive surgery. Longer observation periods may therefore be required for HRV‐based monitoring to capture meaningful patterns of postoperative autonomic recovery. Future studies with larger cohorts, a wider range of surgical procedures, and extended follow‐up are warranted to better clarify the clinical utility of HRV in assessing recovery after thoracic surgery.

## Funding

This study was supported by Tampere Tuberculosis Foundation (12‐1025‐13); Finnish Cultural Foundation (50251204); and Aarne Koskelo Foundation.

## Disclosure

An earlier version of this manuscript was published as a preprint on Research Square: 10.21203/rs.3.rs-3843452/v1. The present manuscript reports a secondary analysis focusing exclusively on patients undergoing thoracic surgery. The cardiac surgery cohort was analyzed separately due to the different nature of the surgical procedures and physiological responses. Although the trial registration allowed inclusion of participants aged 16–18 years, no underaged participants were enrolled in the cohorts analyzed in this study. All included participants were adults. Differences between the registered sample size and the final study population reflect exclusions due to changes in surgical plans and incomplete data.

## Ethics Statement

In this study, all procedures performed in human participants were in accordance with the ethical standards of the institutional and national research committees and with the 1964 Helsinki Declaration and its later amendments or comparable ethical standards. Written informed consent was obtained from all individual participants included in the study.

## Conflicts of Interest

The authors declare no conflicts of interest.

## Supporting information


**Supporting Information** Additional supporting information can be found online in the Supporting Information section. Table S1: Classification of surgical procedures included in the study, grouped into pulmonary resection (PR, major surgery) and minor pulmonary (PM, minor surgery) categories. The table summarizes representative surgical procedures within each group and indicates the typical surgical approach (video‐assisted thoracoscopic surgery [VATS] and/or thoracotomy) associated with each procedure type.

## Data Availability

The data that support the findings of this study are available on request from the corresponding author. The data are not publicly available due to privacy or ethical restrictions.
