# Supplementary material for: Quantification of Immediate Surgical Stress After Major and Minor Thoracic Surgeries Through HRV: A Secondary Post Hoc Analysis of a Randomized Controlled Trial
Source: Pulm Med. 2026 Sep 30;2026:3752161. doi: 10.1155/pm/3752161 (PMC13625828; doi:10.1155/pm/3752161)
Supplement: Supplementary file 1 [file PM-2026-3752161-s001.docx]

**Supplementary Table S1.** Classification of surgical procedures included in the study, grouped into pulmonary resection (PR, major surgery) and minor pulmonary (PM, minor surgery) categories. The table summarizes representative surgical procedures within each group and indicates the typical surgical approach (video-assisted thoracoscopic surgery [VATS] and/or thoracotomy) associated with each procedure type.

| **Category** | **Procedure** | **Surgical Approach** |
| --- | --- | --- |
| PR (major) | Lobectomy | VATS / Thoracotomy |
| PR (major) | Bilobectomy | VATS / Thoracotomy |
| PR (major) | Pneumonectomy | Thoracotomy |
| PM (minor) | Wedge resection | VATS / Thoracotomy |
| PM (minor) | Lung biopsy | VATS / Thoracotomy |
| PM (minor) | Excision / enucleation | VATS / Thoracotomy |
| PM (minor) | Pleural procedures | VATS |
